# A comprehensive descriptive assessment of obesity related chronic morbidity and estimated annual cost burden from a population-based electronic health record database

**DOI:** 10.1186/s13584-020-00378-1

**Published:** 2020-06-24

**Authors:** Orna Reges, Morton Leibowitz, Avital Hirsch, Dror Dicker, Nick Finer, Christiane Lundegaard Haase, Altynai Satylganova, Maya Leventer-Roberts, Becca Feldman

**Affiliations:** 1 Clalit Research Institute, Clalit Health Services, 2 Shoham, 5251003 Ramat Gan, Israel; 2 Department of Health Systems Management, Ariel University, Ariel, Israel; 3 Department of Preventive Medicine, Feinberg School of Medicine, Northwestern University, Chicago, IL USA; 4 Internal Medicine Department D and EASO Collaborating Center for Obesity Management, Hasharon Hospital, Rabin Medical Center, Keren Kayemet Leyisrael 7, Petach Tikva, 4937211 Israel; 5 Sackler School of Medicine, Tel Aviv University, Tel Aviv, Israel; 6 Bariatric Center, Herzliya Medical Center, Herzliya, Israel; 7 Global Medical Affairs Management, Novo Nordisk A/S, Vandtårnsvej 114, Søborg, DK-2860 Denmark; 8 Global Patient Access, Novo Nordisk A/S, Vandtårnsvej 114, DK-2800 Søborg, Denmark

**Keywords:** Obesity, Morbidity, Cost burden

## Abstract

**Background:**

The growing prevalence of obesity and its complications pose a huge burden on the individual and health care systems worldwide. This study presents the frequency of multiple prevalent co-morbidities and estimated annual cost burden by body mass index (BMI) groups, age, and sex among the Israeli adult population to provide policy makers with further evidence to appropriately target interventions.

**Methods:**

This cross-sectional study utilized population-based electronic medical records from the largest payer-provider health fund in Israel. The population included individuals ≥25 years as of 01/01/2014. A new approach assessing body system-related morbidity (BSRM) prevalence was assessed along with estimated annual cost burden for the year 2015 and presented across BMI group, age, and sex via heat maps.

**Results:**

Among 1,756,791 adults, 65% had an elevated BMI (BMI > 25 kg/m^2^). Heat map analysis demonstrated a higher multi-BSRM prevalence and relative estimated annual cost burden among participants with obesity in all age groups. There was a notably higher multi-BSRM prevalence among men and women aged 25–29 with class III obesity (26 and 30%, respectively) compared to the corresponding BMI groups between 18·5- < 25 kg/m^2^ (5 and 9%, respectively). Healthcare costs were 1·72 times higher among men aged 25–29 with class III obesity and 2·75 times among women aged 25–29 with class III obesity compared to those of healthy weight.

**Conclusions:**

The detailed analysis describes the uneven distribution of burdens across BMI groups, age, and sex allowing policy makers to identify sub-populations for targeted interventions.

## Background

Over the last few decades, an increase in overweight and obesity has been observed in many countries globally [1]. The growing prevalence of obesity and its complications impose huge burdens for the individual and for society and they threaten to overwhelm national health care systems [2–6]. To fully understand this phenomenon and to understand any potential economic benefit from prevention and treatment, a comprehensive picture of costs and co-morbidities needs to be developed. The advent of electronic health records has allowed detailed analysis of health care burdens both globally and in specific sub-segments of the population. Large-scale real-world databases that integrate both clinical and claims data enable researchers and policy makers to look at the prevalence of obesity, and to describe the broad array of co-morbidities and costs across BMI groups.

A recent comprehensive analysis assessed twenty co-morbidities that have a strong association with obesity, and demonstrated an upward global trend in obesity and its associated disease burden [7]. An accompanying editorial stressed the need for continuous surveillance, and the creation of cohorts for the study of obesity prevalence and its associated chronic diseases [8]. While previous studies have presented co-morbidity burdens among the population with obesity, their results were not generalizable due to limited sample sizes or overly specific sample selection criteria [9, 10].

In addition to documented co-morbidities, studies have also demonstrated that BMI is directly associated with healthcare cost [11, 12]. A study by Li et al. [11] (2015) assessed the economic burden of 21 obesity-related disorders among 56,895 subjects and found that the costs associated with one prevalent obesity related condition (ORC), when all other ORCs were adjusted for, ranged from $120 (for angina) to $1665 (for pulmonary embolism) per person per year. In addition, a study by Attella et al. [12] evaluated net health costs (the sum of indirect and direct costs) among 557,145 individuals, based on seven obesity-related chronic diseases across BMI groups, and demonstrated a J-shape association between BMI and net health costs. The magnitude of this healthcare cost and how co-morbidities distribute across the population with obesity is unknown. A comprehensive assessment of the obesity health care burden across the full spectrum of BMI groupings is missing. Health care organizations can use detailed data to direct cost-effective management policies to improve access to treatment and bring health gains and resource savings. The objective of the present study was to describe obesity related chronic morbidity and estimated annual cost burden among the Israeli population using a large population-based database, and to create a visual representation of the distribution of burdens across BMI groups.

## Methods

### Setting

In Israel, all citizens receive full health care coverage by participation in any one of four integrated payer-provider health funds. Clalit Health Services (Clalit) is the largest of the four with more than 50% of the population and a low member attrition rate of less than 2%. Since 2002, all health care records are electronic and available in a data warehouse for research purposes. This study used retrospective data recorded in Clalit data warehouse. The data warehouse includes socio-demographic information, along with data recorded in inpatient and outpatient encounters, clinical markers, laboratory and imaging tests, and all medications prescribed and dispensed. Recording of BMI has been a quality measure in the system since 2008 and it is routine practice for primary care physicians to record adult patients’ height and weight every two to three years (for adults aged 20–74 and ≥ 75, respectively).

### Study design and population

This was a descriptive cross-sectional study that identified the study population as of 01 January 2014 (index date). The study population consisted of Clalit members who were aged 25 or older as of the index date with at least one documented BMI during the 3 years previous to the index date. Individuals with no recorded BMI were excluded. Patients were further excluded from the study if they met one or more of the following criteria: their last recorded BMI in the previous 3 years was < 18·5 kg/m^2^ (underweight); they were not continuous members of Clalit during the 3 years previous to the index date through to 1 year following the index date (allowing for death occurring during 2014); and women whose recorded BMIs during the 3 years all occurred during a documented pregnancy.

### Variables

BMI (kg/m^2^) level was determined based on the last height and weight measurement recorded during the 3 years prior to the index date. BMI was grouped into five categories: healthy weight (18·5- < 25 kg/m^2^), overweight (25- < 30 kg/m^2^), class I obesity (30- < 35 kg/m^2^), class II obesity (35- < 40 kg/m^2^), and class III obesity (≥40 kg/m^2^).

Socio-demographic characteristics were assessed at the index date as follows: age (in years and grouped into 6 categories: 25–29, 30–39, 40–49, 50–59, 60–69, and ≥ 70), sex, immigrant status (yes, no), years in country among immigrants, ethnicity (Israeli-born Jewish, European-born Jewish; Middle Eastern-born Jewish; Americas-born Jewish; former USSR-born Jewish; Ethiopian-born Jewish; Arab; missing), socio-economic status (low, medium, or high), is based on an individual’s postal code, that is based on a modified version the Characterization and Classification of Geographical Units by the Socio-Economic Level of the Population as defined/published by the Israel Bureau of Statistics [13]. If that data is missing, an ecological variable is used which is based on the patient’s socio-economic status assigned to their primary care clinic.

Age and BMI group was defined for each individual based on the categorial variables (30 categories in total).

Thirty six co-morbidities were evaluated as of the index date based on international classification of diseases, ninth revision, clinical modification codes extracted from both hospital and outpatient diagnoses. These codes are presented in Supplementary Table 1. Based on these co-morbidities, eight body system-related morbidity (BSRM) categories were newly defined in accord with the international classification of diseases taxonomy: metabolic disorders; cardiovascular disease; digestive system and related disorders; urinary system disorders; respiratory disorders; neuropsychological disorders; musculoskeletal disorders; and malignancies. A BSRM was considered to be present if there was a documentation of one or more related co-morbidities anytime between 2002 and the index date. A count variable was calculated for each individual as the total number of BSRM (ranging from 0 to 8). Individuals were further defined as multi-BSRM if they were grouped into at least two BSRM groups. In addition, a Charlson co-morbidity index was calculated for each individual [14].

Annual healthcare cost burden (hereby referred to as annual cost burden) was estimated using a Clalit cost compendium, which is a summation of the reported patient-specific total costs associated with the use of all inpatient and outpatient visits, procedures, diagnoses, medication purchases, and laboratory tests.

This study was approved by Clalit’s institutional review board.

### Statistical analysis

Frequency of socio-demographic characteristics, co-morbidities, and BSRM categories were generated across the total study population and by BMI groups. In parallel to the calculation of the mean number of BSRM categories across BMI groups, the mean Charlson co-morbidity index was also calculated across these groups as a validation of this novel grouping of diseases**.** The correlation between the mean number of BSRM and the mean Charlson co-morbidity index was calculated by generating a Pearson correlation coefficient.

Poisson regression was employed to assess the independent prevalence ratio (PR) and 95% confidence interval (CI) for the association between age and BMI group with the number of BSRM. A linear model was used to assess the independent association of age and BMI group with annual healthcare cost burden. The log(x + 1) transform was used to make annual healthcare cost burden conform more closely to the normal distribution. These two multivariate models included sex, SES, and ethnicity in addition to the age and BMI group. Individuals aged 25 to 29 with a healthy weight were defined as the reference groups. All models’ assumptions were tested and fulfilled for all analyses. All statistical tests were two-tailed. Results were considered statistically significant if *p* < .05. Test were conducted using R version 3.5.2.

As a tool for policy makers, two separate heat maps were used for visual representations of the prevalence of multi-BSRM, as well as the relative estimated annual cost burden by sex, age, and BMI group. Table 1 depicts the prevalence of multi-BSRM across the groups as follows: green (prevalence ≤20%), yellow (> 20–40%), orange (> 40–60%), bright red (> 60–80%), and dark red (> 80%).The prevalence of multi-BSRM by age and BMI groups was also described for SES strata (Supplementary Heat map 1). Table 2 depicts the relative estimated annual health cost burden per capita compared to reference group. Males and females aged 25 to 29 with a healthy weight were defined as the reference groups. The groups were divided into quintiles and presented as follows: the lowest quintile of relative cost per capita is displayed in green, followed by yellow, orange, bright red, and dark red as the top quintile.

**Table 1:**
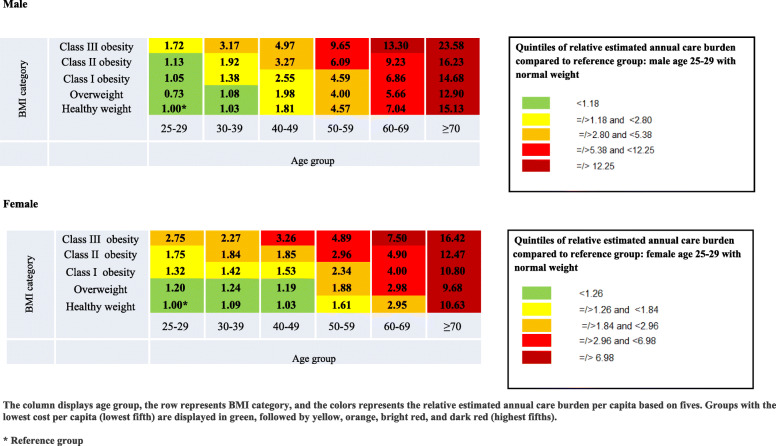
Heat map 1: Relative estimated annual care burden per capita according age and BMI categories as of 01 January 2014

**Table 2:**
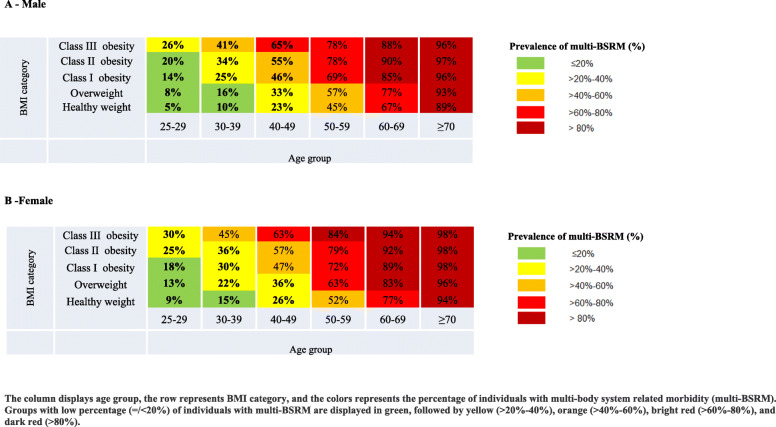
Heat map 2: Prevalence of individuals with multi-body system related morbidity (multi-BSRM) as of 01 January 2014

## Results

There were 2,235,385 Clalit members aged 25 or older as of index date **(**01 January 2014). Supplementary Fig. 1 describes the distribution of this population by BMI category. Individuals with BMI < 18·5 (35,012) and individuals with missing BMI measurement as of index date (441,582) were excluded from the analysis. BMI documentation was missing for 441,582 individuals (20% of eligible patients) who did not have a doctor visit during the 3 years prior to index date. Compared to the total study population, individuals with missing BMI measurements were of younger age (38·88 ± 12·03 years vs. 54·57 ± 17·32 years) and more likely to be male (56·1% vs. 49·2%). Of 1,756,791 members who met the inclusion criteria, 619,504 (35%) had BMI between 18·5 and < 25 kg/m^2^, 661,131 (38%) had BMI between 25 and < 30 kg/m^2^, 323,094 (18%) had BMI between 30 and < 35 kg/m^2^, 106,917 (6%) had BMI between 35 and < 40 kg/m^2^, and 46,145 (3%) had BMI **≥** 40 kg/m^2^ (Table 3). These proportions varied by age with an increase in the percentage of individuals with elevated BMI as age increased (Supplementary Fig. 2).

**Table 3:** Socio-demographic characteristics of the study population as of 01 January 2014

	Normal weight	Overweight	Class I obesity	Class II obesity	Class III obesity	Total patients with elevated BMI	Total Study Population
**BMI range based on last measurement in 2011–2013 (kg/m**^**2**^**)**	**18.5–< 25**	**25- < 30**	**30- < 35**	**35- < 40**	**≥ 40**	**25- ≥ 40**	
Total, N (%)	619,504 (35%)	661,131 (38%)	323,094 (18%)	106,917 (6%)	46,145 (3%)	1,137,287 (65%)	1,756,791 (100%)
Mean (SD)	22.5 (1.7)	27.3 (1.4)	32.1 (1.4)	37.0 (1.4)	44.7 (6.1)	30.3 (4.7)	27.5(5.4)
Median (IQR)	22.8 (21.3–24.0)	27.2 (26.1–28.5)	31.9 (30.9–33.2)	36.8 (35.8–38.1)	42.9 (41.1–45.9)	29.1 (26.9–32.3)	26.7 (23.8–30.4)
Range	18.50–24.99	25.00–29.99	30.00–34.99	35.00–39.99	40.00–141.98	25.00–141.98	18.50–141.98
**Years with current BMI category,** n (%)							
< 5	74,851 (12.1)	134,572 (20.4)	97,032 (30.0)	42,796 (40.0)	15,878 (34.4)	290,278 (25.5)	365,129 (20.8)
> 5	250,773 (40.5)	291,544 (44.1)	136,654 (42.3)	39,503 (36.9)	20,399 (44.2)	488,100 (42.9)	738,873 (42.1)
Unknown	293,880 (47.4)	235,015 (35.5)	89,408 (27.7)	24,618 (23.0)	9868 (21.4)	358,909 (31.6)	652,789 (37.2)
**Time between last measurement of BMI and 1 January 2014 (days)**							
Mean (SD)	358 (291)	297 (259)	262 (237)	241 (225)	232 (220)	279 (220)	306.7 ± 267.47
Median (IQR)	268 (129–534)	225 (99–392)	202 (83–339)	184 (73–318)	177 (70–309)	212 (89–359)	230.0 (101.0–416.0)
Range	0–1096	0–1096	0–1096	0–1096	0–1095	0–1096	0–1096
**Age (years)**							
Mean (SD)	50.35 (18.34)	56.35 (16.76)	57.83 (15.74)	57.42 (15.36)	56.29 (15.03)	56.87 (16.29)	54.57 (17.32)
Median (IQR)	48.0 (34.0–64.0)	57.0 (43.0–68.0)	58.0 (46.0–69.0)	58.0 (46.0–68.0)	57.0 (45.0–67.0)	58.0 (44.0–68.0)	55.0 (40.0–67.0)
Age group, n (%)							
25–34	160,799 (26.0)	80,627 (12.2)	27,726 (8.6)	8932 (8.4)	4172 (9.0)	121,457 (10.7)	282,256 (16.1)
35–44	116,370 (18.8)	101,712 (15.4)	44,395 (13.7)	14,739 (13.8)	6736 (14.6)	167,582 (14.7)	283,952 (16.2)
45–54	91,134 (14.7)	110,977 (16.8)	58,507 (18.1)	20,727 (19.4)	9257 (20.1)	199,468 (17.5)	290,602 (16.5)
55–64	103,812 (16.8)	150,576 (22.8)	80,038 (24.8)	26,786 (25.1)	12,022 (26.1)	269,422 (23.7)	373,234 (21.2)
65–74	69,352 (11.2)	111,123 (16.8)	59,669 (18.5)	19,584 (18.3)	8208 (17.8)	198,584 (17.5)	267,936 (15.3)
75–84	49,489 (8.0)	75,841 (11.5)	39,718 (12.3)	12,720 (11.9)	4656 (10.1)	132,935 (11.7)	182,424 (10.4)
85+	28,548 (4.6)	30,275 (4.6)	13,041 (4.0)	3429 (3.2)	1094 (2.4)	47,839 (4.2)	76,387 (4.3)
**Sex,** n (%)							
Male	298,452 (48.2)	367,837 (55.6)	148,931 (46.1)	36,411 (34.1)	11,994 (26.0)	565,173 (49.7)	863,625 (49.2)
Female	321,052 (51.8)	293,294 (44.4)	174,163 (53.9)	70,506 (65.9)	34,151 (74.0)	572,114 (50.3)	893,166 (50.8)
**Immigrant,** n (%)							
Yes	210,409 (34.0)	263,428 (39.8)	131,586 (40.7)	41,936 (39.2)	16,730 (36.3)	453,680 (39.9)	664,089 (37.8)
No	409,095 (66.0)	397,703 (60.2)	191,508 (59.3)	64,981 (60.8)	29,415 (63.7)	683,607 (60.1)	1,092,702 (62.2)
**Years in country (among foreign born)**							
Mean (SD)	42 (19.99)	44.65 (18.98)	44.2 (18.66)	42.75 (18.46)	41.11 (18.29)	44.21 (18.83)	43.52 (19.23)
Median (IQR)	45.0 (23.0–63.0)	50.0 (24.0–63.0)	50.0 (24.0–63.0)	46.0 (24.0–59.0)	42.0 (23.0–58.0)	50.0 (24.0–63.0)	49.0 (24.0–63.0)
**Years in country (among foreign born)**							
**Ethnicity,** n (%)							
Israeli-born Jewish	319,949 (51.6)	278,142 (42.1)	118,311 (36.6)	36,993 (34.6)	15,756 (34.1)	449,202 (39.5)	769,151 (43.8)
European-born Jewish	38,387 (6.2)	50,007 (7.6)	22,728 (7.0)	6612 (6.2)	2269 (4.9)	81,616 (7.2)	120,003 (6.8)
Middle Eastern-born Jewish	34,244 (5.5)	43,572 (6.6)	19,218 (5.9)	5047 (4.7)	1627 (3.5)	69,464 (6.1)	103,708 (5.9)
Americas-born Jewish	11,320 (1.8)	12,405 (1.9)	5951 (1.8)	1884 (1.8)	788 (1.7)	21,028 (1.8)	32,348 (1.8)
Former USSR-born Jewish	64,774 (10.5)	81,945 (12.4)	46,231 (14.3)	16,376 (15.3)	7272 (15.8)	151,824 (13.3)	216,598 (12.3)
Ethiopian-born Jewish	17,946 (2.9)	10,556 (1.6)	2571 (0.8)	442 (0.4)	97 (0.2)	13,666 (1.2)	31,612 (1.8)
Arab	91,103 (14.7)	122,374 (18.5)	75,087 (23.2)	28,785 (26.9)	14,082 (30.5)	240,328 (21.1)	331,431 (18.9)
Missing data	41,781 (6.7)	62,130 (9.4)	32,997 (10.2)	10,778 (10.1)	4254 (9.2)	110,159 (9.7)	151,940 (8.6)
**Socioeconomic status,** n (%)							
Low	133,860 (21.6)	164,535 (24.9)	96,709 (29.9)	36,542 (34.2)	17,629 (38.2)	315,415 (27.7)	449,275 (25.6)
Medium	241,575 (39.0)	261,478 (39.6)	129,928 (40.2)	43,110 (40.3)	18,337 (39.7)	452,853 (39.8)	694,428 (39.5)
High	243,481 (39.3)	234,536 (35.5)	96,179 (29.8)	27,190 (25.4)	10,132 (22.0)	368,037 (32.4)	611,518 (34.8)
Missing data	588 (0.1)	582 (0.1)	278 (0.1)	75 (0.1)	47 (0.1)	982 (0.1)	1570 (0.1)
**Residence,** n (%)							
Urban	554,723 (89.5)	593,628 (89.8)	293,270 (90.8)	97,925 (91.6)	42,700 (92.5)	1,027,523 (90.3)	1,582,246 (90.1)
Rural	63,858 (10.3)	66,684 (10.1)	29,503 (9.1)	8882 (8.3)	3423 (7.4)	108,492 (9.5)	172,350 (9.8)
Missing data	923 (0.1)	819 (0.1)	321 (0.1)	110 (0.1)	22 (0.0)	1272 (0.1)	2195 (0.1)
**Number of children**							
Mean (SD)	2.08 ± 1.97	2.50 ± 2.15	2.75 ± 2.35	2.85 ± 2.50	2.85 ± 2.60	2.62 ± 2.26	2.43 ± 2.18
Median (IQR)	2.0 (0.0–3.0)	2.0 (1.0–4.0)	3.0 (1.0–4.0)	3.0 (1.0–4.0)	2.0 (1.0–4.0)	2.0 (1.0–4.0)	2.0 (1.0–3.0)

Subjects with an elevated BMI (> 25 kg/m^2^), compared to patients with healthy weight, were on average of older age (median age 58 vs. 48 years, respectively) and had more children (2·62 vs. 2·08, respectively). The proportion of Arabs and individuals with a low socio-economic status was higher among subjects with an elevated BMI (21·1% vs. 14·7%, respectively and 27·7% vs. 21·6%, respectively). All the comparisons were statistically significant (*p* < 0.05) (Table 3).

Subjects with an elevated BMI (> 25 kg/m^2^) had more co-morbidities compared to those of healthy weight. Significant differences were demonstrated with respect to pre-diabetes, diabetes, ischemic heart disease, atrial fibrillation, ischemic stroke, congestive heart failure, peripheral artery disease, hypertension, dyslipidemia, hyperthyroidism, non-alcoholic fatty liver disease, gastroesophageal reflux disease, gall-bladder disease, urinary incontinence, asthma, chronic obstructive pulmonary disease, and obstructive sleep apnea. The mean number of BSRM categories was also higher among individuals with an elevated BMI (> 25 kg/m^2^) (2·37 ± 1·72) compared to individuals with healthy weight (1·57 ± 1·62) (Supplementary Table 2).

The Poisson regression demonstrated a significant association of age and BMI group with the number of BSRM after adjustment for sex, SES, and ethnicity. Compared to individuals aged 25 to 29 with a healthy weight, the adjusted PR increased as the age and the BMI increased, with the highest prevalence in those aged ≥70 with class III obesity (PR = 9.65, 95%CI: 9.51 to9.79). In the same way, adjusted linear regression showed a significant relationship between age and BMI group with costs (*p* < 0.001). The annual cost increased as both age and BMI increased, with the highest cost among individuals aged ≥70 with class III obesity (exp (β) = 10.70, 95%CI: 10.28 to 11.13) (Table 4).

**Table 4:** Adjusted association of age and BMI group with the number of Body system-related morbidity (BSRM) and annual cost burden

Age and BMI group	BSRM^a^	Annual Cost	
	PR (95% CI)	β (95% CI) ^b^	Exp β (95% CI)^c^
Age 25–29			
Healthy weight	Reference group	Reference group	Reference group
Overweight	1.23 (1.21–1.25)	0.04 (0.02–0.06)	1.04 (1.02–1.06)
Class I obesity	1.65 (1.61–1.69)	0.21 (0.17–0.24)	1.23 (1.19–1.28)
Class II obesity	2.12 (2.05–2.20)	0.44 (0.38–0.50)	1.55 (1.46–1.65)
Class III obesity	2.53 (2.42–2.65)	0.71 (0.62–0.79)	2.03 1.87–2.21)
Age 30–39			
Healthy weight	1.41 (1.40–1.43)	0.08 (0.06–0.09)	1.08 (1.07–1.10)
Overweight	1.85 (1.83–1.87)	0.14 (0.13–0.16)	1.15 (1.13–1.17)
Class I obesity	2.48 (2.44–2.51)	0.36 (0.34–0.39)	1.43 (1.40–1.47)
Class II obesity	2.99 (2.93–3.04)	0.61 (0.58–0.65)	1.84 (1.78–1.91)
Class III obesity	3.52 (3.43–3.61)	0.92 (0.87–0.97)	2.51 (2.38–2.64)
Age 40–49			
Healthy weight	2.26 (2.24–2.29)	0.32 (0.30–0.34)	1.38 (1.35–1.40)
Overweight	2.97 (2.94–3.01)	0.46 (0.44–0.47)	1.58 (1.56–1.61)
Class I obesity	3.74 (3.69–3.78)	0.71 (0.69–0.73)	2.03 (1.99–2.08)
Class II obesity	4.37 (4.30–4.43)	0.94 (0.91–0.97)	2.56 (2.48–2.64)
Class III obesity	4.98 (4.88–5.07)	1.17 (1.13–1.21)	3.22 (3.09–3.36)
Age 50–59			
Healthy weight	3.87 (3.83–3.91)	0.81 (0.80–0.83)	2.25 (2.21–2.29)
Overweight	4.65 (4.59–4.70)	1.02 (1.01–1.04)	2.77 (2.73–2.82)
Class I obesity	5.46 (5.4 0–5.52)	1.24 (1.22–1.26)	3.46 (3.39–3.52)
Class II obesity	6.16 (6.08–6.24)	1.41 (1.39–1.44)	4.10 (3.99–4.20)
Class III obesity	6.72 (6.62–6.83)	1.57 (1.53–1.61)	4.81 (4.64–4.98)
Age 60–69			
Healthy weight	5.54 (5.48–5.60)	1.35 (1.33–1.37)	3.86 (3.79–3.92)
Overweight	6.22 (6.16–6.29)	1.56 (1.54–1.57)	4.76 (4.68–4.83)
Class I obesity	6.98 (6.91–7.06)	1.76 (1.74–1.78)	5.81 (5.71–5.92)
Class II obesity	7.65 (7.55–7.75)	1.91 (1.88–1.94)	6.75 (6,58–6.93)
Class III obesity	8.12 (8.00–8.24)	2.04 (2.00–2.08)	7.69 (7.41–7.98)
Age > =70			
Healthy weight	8.11 (8.02–8.20)	2.12 (2.10–2.13)	8.33 (8.19–8.48)
Overweight	8.52 (8.43–8.61)	2.21 (2.20–2.23)	9.12 (8.97–9.26)
Class I obesity	9.00 (8.90–9.10)	2.30 (2.28–2.32)	9.97 (9.79–10.16)
Class II obesity	9.37 (9.25–9.48)	2.34 (2.31–2.37)	10.38 (10.11–10.66)
Class III obesity	9.65 (9.51–9.79)	2.37 (2.33–2.41)	10.70 (10.28–11.13)

*BSRM* Body system related morbidity, *PR* Prevalence rate, *CI* Confidence interval

^a^The association of age and BMI group with BSRM was assessed by using Poisson regression. The dependent variable was defined as the total number of BSRM (range from 0 to 8). Model adjusted for sex, socioeconomic status (low, medium, and High), and ethnicity (Israeli-born Jewish, European-born Jewish; Middle Eastern-born Jewish; Americas-born Jewish; former USSR-born Jewish; Ethiopian-born Jewish; Arab; missing)

^b^The association of age and BMI group with annual cost burden was assessed by using linear regression. The dependent variable was defined as log(x + 1) transform of the estimated annual cost burden using a Clalit cost compendium. Model adjusted for sex, socioeconomic status (low, medium, and High), and ethnicity (Israeli-born Jewish, European-born Jewish; Middle Eastern-born Jewish; Americas-born Jewish; former USSR-born Jewish; Ethiopian-born Jewish; Arab; missing)

^c^Retransformation adjustment (exp β) was performed in going from the log model to estimates of Clalit cost compendium (NIS)

Tables 1 and 2 depict multi-BSRM prevalence and relative estimated annual cost burden per capita according to age and BMI categories. There was a higher multi-BSRM prevalence and relative estimated annual cost burden among males and females with obesity (BMI ≥ 30) in all age groups. There was a greater BSRM prevalence among younger males and females with obesity class III aged 25 to 29 (26 and 30%, respectively) compared to men and women of the same age group with healthy weight range (5 and 9%, respectively) (Table 1). The higher multi-BSRM prevalence among individuals with obesity in all age groups was preserved also across SES strata (Supplementary Heat map 1). Higher multi-BSRM prevalence was also observed in individuals with overweight (BMI ≥ 25 and < 30) compared to those with healthy weight among males and females in all age groups. Based on the reference group (males and females aged 25–29 at a healthy weight), healthcare costs increased 1·72 times among men aged 25–29 with class III obesity and 2·75 times among women aged 25–29 with class III obesity (Table 2). Multi-BSRM prevalence and relative estimated annual cost burden was highest at the oldest age groups. The highest Multi-BSRM prevalence and cost burden was observed among the oldest population with highest BMI group.

## Discussion

### Main findings

This study used comprehensive electronic health record data from a large, population-based sample in Israel and presents significant associations of BMI groups with the number of BSRM and annual cost burden after adjustment for age, sex, SES, and ethnicity. As a tool for policy makers, a comprehensive picture of the increasing prevalence of BSRM categories and associated costs across BMI groups were depicted by using heat maps. Subjects with an elevated BMI (> 25 kg/m^2^) had more co-morbidities and healthcare costs compared to those with healthy weight. There was a notably high percentage of both young men and women with multi-BSRM among the group with Class III obesity.

### The association of BMI with morbidity & costs

The suggestion that co-morbidities and costs are directly related to BMI level has been demonstrated in previous studies [15–17]. A comprehensive literature review and meta-analysis (2009) related the incidence of 18 co-morbidities to severity of obesity [16]. Booth et al. [9] (2014) also demonstrated an association between prevalence of multi-morbidity and increasing BMI. Their study included 223,089 individuals and presented the prevalence of multi-morbidities by six BMI categories ranging from underweight through healthy weight, overweight, and three stages of increasing BMI stratifying by age and sex. Booth et al.’s study included 11 conditions, affecting seven systems of the body, with multi-morbidity defined as having ≥2 co-morbidities out of the 11. Li et al. [11] used data from the Geisinger System EHRs and examined the additional costs of isolated co-morbidities associated with obesity and the individual cumulative economic impact on the health care system. They demonstrate that the incremental additional cost of hypertension, as a result of its frequency, exceeds the incremental cumulative costs of MI and/or Pulmonary embolus because of their less frequent occurrence. Atella et al. [12] used the EHRs of 700 volunteer general practitioners in Italy to assess the individual risk of multiple co-morbidities as a consequence of the patients’ BMI status. The similarity in the emergence of multiple co-morbidities as a function of both increasing BMI and increasing age parallels the current finding in a broader non-selected assessment of a total population. The current study extends the findings from these large-scale studies by including a larger population size with a greater number of co-morbidities, which enabled us to group chronic conditions into eight comprehensive BSRM categories, along with their distribution across increasing BMI groups. Furthermore, the size of the present study population enabled analyzing the data with greater resolution, stratifying both comorbidities and costs by 60 different strata of BMI, age, and sex groups. The strength of this increased resolution can best be appreciated by the strengths of association presented in the adjusted regression analysis between the numbers of BSRM as well as the related costs in the younger age groups among the overweight and lower classes of obesity (Table 4). We are able to see that the differences at the young ages are modest and yet the differences are significant and become greater as age and BMI increase.

A notable finding from the current study was the percentage of young adults (25–29 years) with multi-BSRM with class III obesity, especially among women, who presented with a higher percentage of co-morbidities compared to men. This finding of multi-morbidity in the younger age group has been previously reported in a general population study [18]. The present study placed emphasis on this increase of multi-morbidity as a function of age, sex, and BMI, by both incorporating a larger array of morbidities and stratifying by BMI. In addition, these observations suggest that in addition to *diabesity* (which refers to diabetes occurring in the framework of obesity), the term *morbesity* can be used as the wide array of co-morbidities associated with obesity among both males and females at all ages.

### Implications for practice (tool for decision makers)

This comprehensive cross-sectional study provides a multi-dimensional template for national health care plans to direct policy among key sub-populations with varying health and economic burdens. The detailed heat maps presented could allow health policy makers to make decisions based on the presented disease and economic burden within and across subgroups based on BMI, age, and sex. Policy makers can utilize the heat maps presented to both select groups for intervention as the obesity burden can be costly to the system, as well as to identify complex patient groups who require specialized care and timely intervention. Such data are potentially of great importance given the size of the obesity problem, and the often high costs of weight loss treatments (e.g., bariatric surgery) [19, 20].

Suggestions for practice could include building outpatient clinics that provide continuous care programs dedicated to comprehensively treating and preventing obesity. These clinics would treat patients with obesity, and provide support and follow-up to help them achieve and maintain a healthy BMI. Interventions include: dietician visits, physical activity, follow-up and screening tests for obesity-related comorbidities, emotional support, and consultations regarding weight-loss medication and bariatric surgery. As informed by our study results, target groups would include individuals with both obesity and multi-BSRM across all age groups, and young adults with obesity without co-morbidities. In addition, individuals with overweight (BMI ≥ 25 and < 30), who have a higher burden of multi-BSRM than those with healthy weight, should be considered as individuals with pre-obesity, and therefore should also be targeted for continuous care programs.

### Limitations

This study had a number of limitations. First, since the analysis was cross sectional in its design, the cause of the morbidities or health cost burden could not be determined. Future longitudinal assessment is needed to evaluate the trajectory of health problems over time and to identify patterns of illness trajectories across BMI categories. Second, this analysis was limited to patients who had at least one BMI record collected by their physician in their health record in the 3 years prior to the index date. Thus, it is possible that we are not capturing individuals who were young and healthy, had low health care utilization, or were likely to have poor access to healthcare services. Third, costs from the Israeli healthcare system reported in this study are particular to Clalit Health Services’ internal pricing scheme, and therefore, the annual cost burden was presented as relative rather than absolute costs. Lastly, although a wide array of diagnoses was included in the analysis, the list may not be exhaustive due to less comprehensive documentation with some co-morbidities.

## Conclusion

This study provides a response to the recognized need for greater resolution in health and economic data when reviewing health care policy. The heat maps presented here describe uneven distribution of burdens across BMI groups, age, and sex. This detailed analysis provides to the public health community an illustration of how electronic health record data may be used to facilitate health policy planning.

## Supplementary information

**Additional file 1: Table S1.** Diagnosis definition/ICD-9-CM diagnoses codes

**Additional file 2: Supplementary Heat map 1.** Prevalence of individuals with multi-body system related morbidity (multi-BSRM) as of 01 January 2014

**Additional file 3: Figure S1.** Clalit members aged 25 or older as of 01 January 2014 by BMI level (*n* = 2,233,385)

**Additional file 4: Figure S2.** Clalit members by age and BMI level (2014)

**Additional file 5: Table S2.** Concurrent co-morbidities of the study population as of 01 January 2014.

## Data Availability

The raw data used for this study will be stored at the Clalit servers and within its firewall, and will be made available upon request under the limitations and requisites of the Clalit regulations and Israeli Privacy Laws.

## Abbreviations

BMI: Body mass index
Clalit: Clalit Health Services
BSRM: Body system-related morbidity
ORC: Obesity related condition
