# Supplementary material for: A comprehensive descriptive assessment of obesity related chronic morbidity and estimated annual cost burden from a population-based electronic health record database
Source: Isr J Health Policy Res. 2020 Jun 24;9:32. doi: 10.1186/s13584-020-00378-1 (PMC7315485; doi:10.1186/s13584-020-00378-1)
Supplement: Supplementary file 1 — Additional file 1: Table S1. Diagnosis definition/ICD-9-CM diagnoses codes [file 13584_2020_378_MOESM1_ESM.docx]

Supplementary Table 1: Diagnosis definition/ICD-9-CM diagnoses codes

| **Diagnosis** | **Definition / ICD-9-CM codes** |
| --- | --- |
| Prediabetes | Modified using the American Diabetes Association (ADA) definition [[5](#_ENREF_5)]: Individuals with at least two of the following community reported laboratory results within a year (during the 3 years prior to index date) were considered as patients with prediabetes: fasting glucose level between 100 and 125; HbA1c level >/= 5.7 and < 6.5. |
| Diabetes | Based on CRI's algorithm [[6](#_ENREF_6)]. |
| Dyslipidemia | 272.0; 272.1; 272.2; 272.4; 272.5; 272.9; 272.0; 272.1; 272.2; 272.4; 272.5; 272.9 |
| Hyperthyroidism | 242.xx |
| Hypothyroidism | 244.xx |
| Acute myocardial infarction (AMI) | 410.x;411.0;412.x |
| Unstable angina pectoris (UAP) | 411; 411.1; 411.8; 411.81; 411.89; 413.0; 413.1 |
| Stable angina pectoris (AP) | 413;413.9 |
| Percutaneous transluminal coronary angioplasty (PTCA) | 36.0x |
| Coronary artery bypass grafting (CABG) | 36.1x;36.3x |
| Ischemic heart disease (IHD) | Any of the above 5 heart diagnoses/interventions (i.e. AMI, UAP, AP, PTCA, and CABG) or ICD-9-CM codes: 414.xx, 429.2 |
| Atrial fibrillation | 427.31-3 |
| Ischemic stroke | 433.x1, 434.x1, 436, 437.1, 437.9 |
| Congestive heart failure | 398.91, 402.01, 402.11, 402.91, 404.01, 404.03, 404.11, 404.13, 404.91, 404.93, 425.4–425.9, 428.x |
| Pulmonary embolism | 415.1 |
| Peripheral artery disease | 443.9 |
| Hypertension | 401.xx-405.xx |
| Non-alcoholic fatty liver disease (NAFLD) | 571.8 |
| Gastroesophageal reflux disease (GERD) | 530.81 |
| Gall bladder disease (GBD) | 574.xx, 575.xx |
| Urinary incontinence | 788.3x |
| Chronic Kidney Disease (CKD) | CKD was classified into the following stages:   - Stage 1(CKD1): GFR>/=90.0mL/min/1.73 m2; - Stage 2 (CKD2): GFR >/=60.0 to <90 mL/min/1.73 m2; - Stage 3A (CKD3A): GFR >/=45 to <60 mL/min/1.73 m2; - Stage 3B (CKD3B): GFR >/=30 to< 45 mL/min/1.73 m2; - Stage 4 (CKD4): GFR >/=15 to <30 mL/min/1.73 m2; - Stage 5 (CKD5): GFR < 15 mL/min/1.73 m2   Renal replacement therapy (RRT): any documentation of end stage renal disease, kidney failure, dialysis, or renal transplant. |
| Asthma | 493.xx |
| Chronic obstructive pulmonary disease (COPD) | 491.x, 492.x, 496.x |
| Obstructive sleep apnea (OSA) | 327.23, 780.57 |
| Depression | At least one purchase of an anti-depressant medication [Anatomical Therapeutic Chemical (ATC): N06A, N06CA] |
| Osteoporosis | 733.00 |
| Osteoarthritis | 715.xx, 721.0, 721.2, 721.3, 721.90 |
| Active malignancy: Any type of cancer | A member was considered to have an active malignancy if one or more of the following are recorded **within five years prior to index date**:   - Israeli cancer registry - Radiation therapy - Metastases - Medical procedures associated with cancer treatment - Medication associated with cancer treatment, based on ATC5 codes - Two Clalit registries: chronic and oncologic, with internal codes and SEER-based coding. |
| Breast cancer (ever) | As defined under “active malignancy,” with no time limitation. |
| Colorectal cancer (ever) | As defined under “active malignancy,” with no time limitation. |
|  |  |
| Pancreatic cancer (ever) | As defined under “active malignancy,” with no time limitation. |
| Endometrial cancer (ever) | As defined under “active malignancy,” with no time limitation. |
| Ovarian cancer (ever) | As defined under “active malignancy,” with no time limitation. |
| Esophageal cancer (ever) | As defined under “active malignancy,” with no time limitation. |
| Kidney cancer (ever) | As defined under “active malignancy,” with no time limitation. |
