# Supplementary material for: A comprehensive descriptive assessment of obesity related chronic morbidity and estimated annual cost burden from a population-based electronic health record database
Source: Isr J Health Policy Res. 2020 Jun 24;9:32. doi: 10.1186/s13584-020-00378-1 (PMC7315485; doi:10.1186/s13584-020-00378-1)
Supplement: Supplementary file 2 — Additional file 2: Supplementary Heat map 1. Prevalence of individuals with multi-body system related morbidity (multi-BSRM) as of 01 January 2014 [file 13584_2020_378_MOESM2_ESM.docx]

**Supplementary** **Heat map 1: Prevalence of individuals with multi-body system related morbidity (multi-BSRM) as of 01 January 2014**

| BMI category | Class III obesity | **27%** | **41%** | **63%** | 81% | 91% | 97% |
| --- | --- | --- | --- | --- | --- | --- | --- |
|  | Class II obesity | **19%** | **32%** | **54%** | 76% | 90% | 97% |
|  | Class I obesity | **13%** | **25%** | **44%** | 68% | 85% | 96% |
|  | Overweight | **8%** | **16%** | **32%** | 57% | 77% | 92% |
|  | Healthy weight | **5%** | **11%** | **23%** | 47% | 67% | 88% |
|  |  | 25-29 | 30-39 | 40-49 | 50-59 | 60-69 | ≥70 |
|  |  | Age group | | | | | |

**A – Low Socio-Economic Status**

**Prevalence of multi-BSRM (%)**
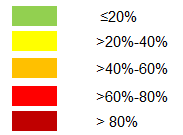


**B -Medium/High Socio-Economic Status**

| BMI category | Class III obesity | **29%** | 45% | 65% | 83% | 93% | 98% |
| --- | --- | --- | --- | --- | --- | --- | --- |
|  | Class II obesity | **25%** | **37%** | 57% | 80% | 92% | 98% |
|  | Class I obesity | **17%** | **29%** | 48% | 72% | 88% | 97% |
|  | Overweight | **11%** | **19%** | **35%** | 61% | 81% | 95% |
|  | Healthy weight | **8%** | **13%** | **25%** | 50% | 73% | 93% |
|  |  | 25-29 | 30-39 | 40-49 | 50-59 | 60-69 | ≥70 |
|  |  | Age group | | | | | |

**Prevalence of multi-BSRM (%)**
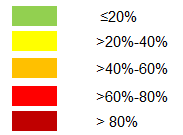


**The column displays age group, the row represents BMI category, and the colors represents the percentage of individuals with multi-body system related morbidity (multi-BSRM). Groups with low percentage (=/<20%) of individuals with multi-BSRM are displayed in green, followed by yellow (>20%-40%), orange (>40%-60%), bright red (>60%-80%), and dark red (>80%).**
