# Supplementary figures and images for: A comprehensive descriptive assessment of obesity related chronic morbidity and estimated annual cost burden from a population-based electronic health record database
Source: Isr J Health Policy Res. 2020 Jun 24;9:32. doi: 10.1186/s13584-020-00378-1 (PMC7315485; doi:10.1186/s13584-020-00378-1)

**Supplementary Figure 1: Clalit members aged 25 or older as of 01 January 2014 by BMI level (n=2,233,385)**


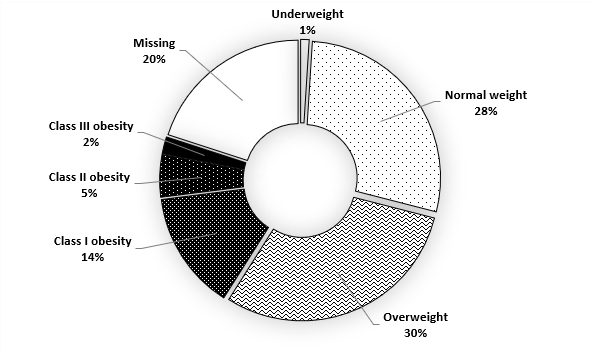

Supplement: Supplementary file 3 — Additional file 3: Figure S1. Clalit members aged 25 or older as of 01 January 2014 by BMI level (n = 2,233,385) [file 13584_2020_378_MOESM3_ESM.docx]
