# Supplementary material for: A comprehensive descriptive assessment of obesity related chronic morbidity and estimated annual cost burden from a population-based electronic health record database
Source: Isr J Health Policy Res. 2020 Jun 24;9:32. doi: 10.1186/s13584-020-00378-1 (PMC7315485; doi:10.1186/s13584-020-00378-1)
Supplement: Supplementary file 4 — Additional file 4: Figure S2. Clalit members by age and BMI level (2014) [file 13584_2020_378_MOESM4_ESM.pdf]

Supplementary Figure 2: Clalit members by age and BMI level (2014)

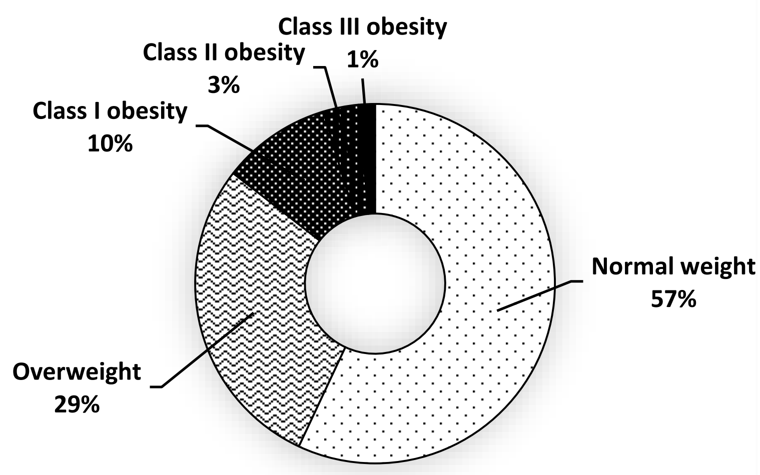

Age group 25-34 (n=282,256)

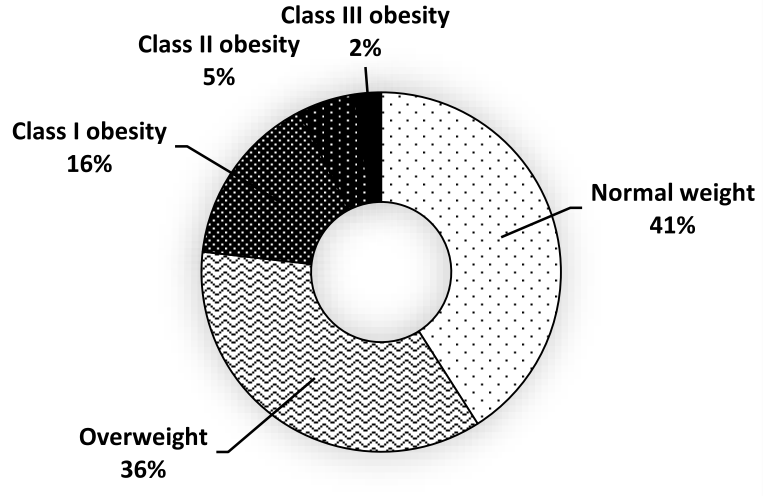

Age group 35-44 (n=283,952)

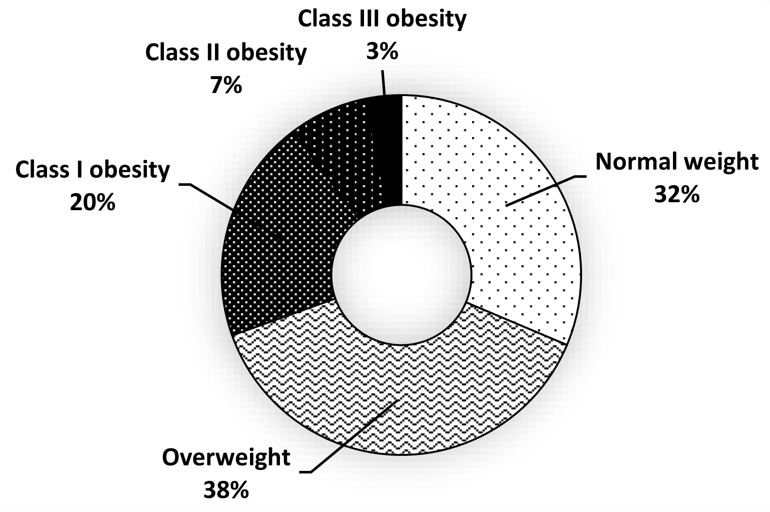

Age group 45-54 (n=290,602)

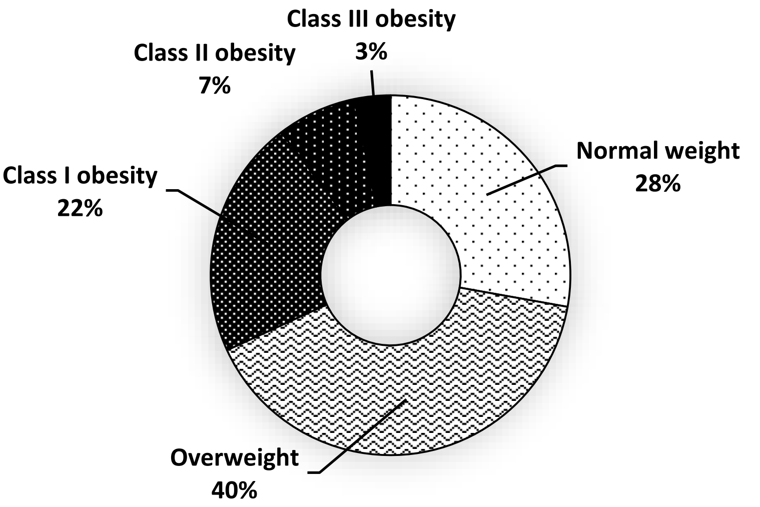

Age group 55-64 (n=373,234)

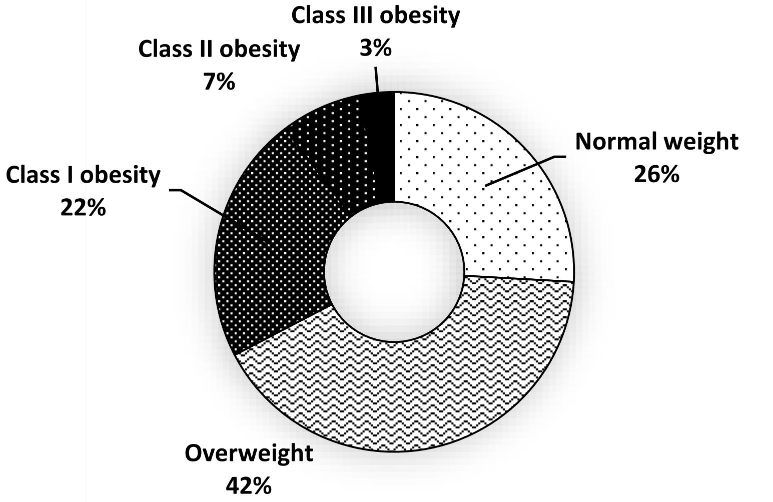

Age group 65-74 (n=267,936)

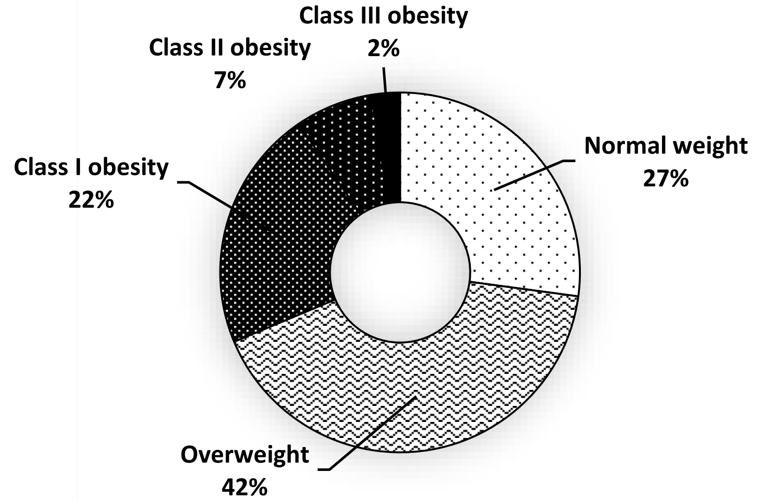

Age group 75-84 (n=182,424)

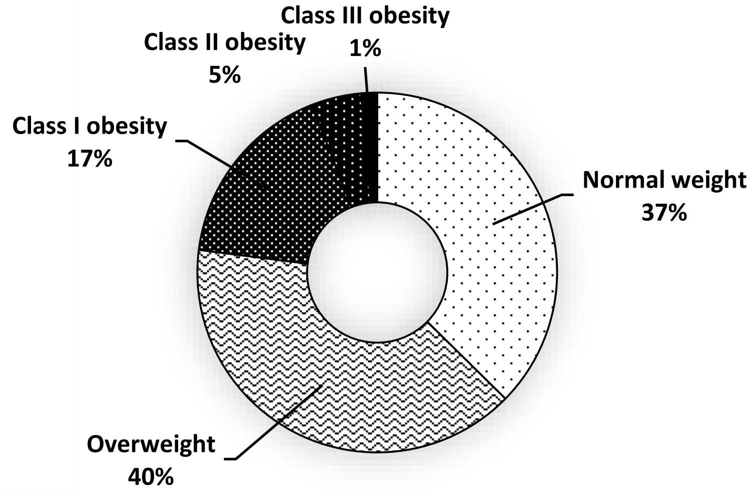

Age group ≥85 (n=76,387)
