# Supplementary material for: A comprehensive descriptive assessment of obesity related chronic morbidity and estimated annual cost burden from a population-based electronic health record database
Source: Isr J Health Policy Res. 2020 Jun 24;9:32. doi: 10.1186/s13584-020-00378-1 (PMC7315485; doi:10.1186/s13584-020-00378-1)
Supplement: Supplementary file 5 — Additional file 5: Table S2. Concurrent co-morbidities of the study population as of 01 January 2014. [file 13584_2020_378_MOESM5_ESM.docx]

**Supplementary Table 2: Concurrent co-morbidities of the study population as of 01 January 2014**

|  | **Normal weight** | **Overweight** | **Class I obesity** | **Class II obesity** | **Class III obesity** | **Total patients with elevated BMI** | **Total study population** |
| --- | --- | --- | --- | --- | --- | --- | --- |
| **BMI (kg/m^2^)** | 18.5-<25 | 25-<30 | 30-<35 | 35-<40 | ≥ 40 | 25-≥ 40 | ≥18.5 |
| **Total** | **619 504** | **661 131** | **323 094** | **106 917** | **46 145** | **1 137 287** | **1 756 791** |
| **Metabolic disorders,** n (%) |  |  |  |  |  |  |  |
| Pre diabetes | 64 720 (10.4) | 105 876 (16.0) | 59 605 (18.4) | 20 095 (18.8) | 8 626 (18.7) | 194 202 (17.1) | 258 922 (14.7) |
| Diabetes | 61 012 (9.8) | 136 023 (20.6) | 100 071 (31.0) | 41 033 (38.4) | 19 667 (42.6) | 296 794 (26.1) | 357 806 (20.4) |
| Dyslipidemia | 240 662 (38.8) | 381 644 (57.7) | 211 259 (65.4) | 71 495 (66.9) | 29 875 (64.7) | 694 273 (61.0) | 934 935 (53.2) |
| Hyperthyroidism | 15 334 (2.5) | 17 696 (2.7) | 9 794 (3.0) | 3 555 (3.3) | 1 574 (3.4) | 32 619 (2.9) | 47 953 (2.7) |
| Hypothyroidism | 55 346 (8.9) | 66 138 (10.0) | 38 226 (11.8) | 14 751 (13.8) | 6 938 (15.0) | 126 053 (11.1) | 181 399 (10.3) |
| **Any of the above metabolic disease** | **293 357 (47.4)** | **439 082 (66.4)** | **244 224 (75.6)** | **84 532 (79.1)** | **36 864 (79.9)** | **804 702 (70.8)** | **1 098 059 (62.5)** |
| **Cardiovascular disease (CVD),** n (%) |  |  |  |  |  |  |  |
| Myocardial infarction | 21 756 (3.5) | 38 597 (5.8) | 20 670 (6.4) | 6 097 (5.7) | 2 217 (4.8) | 67 581 (5.9) | 89 337 (5.1) |
| Unstable angina | 15 065 (2.4) | 28 948 (4.4) | 16 782 (5.2) | 5 407 (5.1) | 2 105 (4.6) | 53 242 (4.7) | 68 307 (3.9) |
| Stable angina | 16 554 (2.7) | 33 101 (5.0) | 19 975 (6.2) | 6 755 (6.3) | 2 655 (5.8) | 62 486 (5.5) | 79 040 (4.5) |
| Angioplasty (PTCA) | 21 976 (3.5) | 41 464 (6.3) | 22 622 (7.0) | 6 787 (6.3) | 2 351 (5.1) | 73 224 (6.4) | 95 200 (5.4) |
| Coronary artery bypass graft | 10 403 (1.7) | 18 659 (2.8) | 9 648 (3.0) | 2 728 (2.6) | 886 (1.9) | 31 921 (2.8) | 42 324 (2.4) |
| Ischemic heart disease (IHD) | 51 999 (8.4) | 94 359 (14.3) | 53 867 (16.7) | 17 661 (16.5) | 7 185 (15.6) | 173 072 (15.2) | 225 071 (12.8) |
| Atrial fibrillation | 19 869 (3.2) | 31 406 (4.8) | 19 382 (6.0) | 7 332 (6.9) | 3 413 (7.4) | 61 533 (5.4) | 81 402 (4.6) |
| Ischemic stroke | 31 025 (5.0) | 45 360 (6.9) | 24 991 (7.7) | 8 270 (7.7) | 3 259 (7.1) | 81 880 (7.2) | 112 905 (6.4) |
| Congestive heart failure | 16 722 (2.7) | 27 002 (4.1) | 18 635 (5.8) | 7 983 (7.5) | 4 433 (9.6) | 58 053 (5.1) | 74 775 (4.3) |
| Pulmonary embolism | 2 175 (0.4) | 3 330 (0.5) | 2 260 (0.7) | 1 054 (1.0) | 643 (1.4) | 7 287 (0.6) | 9 462 (0.5) |
| Peripheral artery disease | 23 669 (3.8) | 36 238 (5.5) | 20 615 (6.4) | 7 116 (6.7) | 3 192 (6.9) | 67 161 (5.9) | 90 830 (5.2) |
| Hypertension | 135 541 (21.9) | 256 027 (38.7) | 165 270 (51.2) | 62 262 (58.2) | 28 602 (62.0) | 512 161 (45.0) | 647 702 (36.9) |
| **Any of the above CVD disease** | **166 739 (26.9)** | **293 596 (44.4)** | **180 749 (55.9)** | **66 445 (62.1)** | **30 073 (65.2)** | **570 863 (50.2)** | **737 602 (42.0)** |
| **Digestive system and related disorders,** n (%) |  |  |  |  |  |  |  |
| Non-alcoholic fatty liver disease (NAFLD) | 11 807 (1.9) | 36 430 (5.5) | 30 105 (9.3) | 12 614 (11.8) | 5 937 (12.9) | 85 086 (7.5) | 96 893 (5.5) |
| Gastroesophageal reflux disease (GERD) | 68 251 (11.0) | 96 204 (14.6) | 50 343 (15.6) | 16 581 (15.5) | 6 546 (14.2) | 169 674 (14.9) | 237 925 (13.5) |
| Gall bladder disease (GBD) | 30 374 (4.9) | 48 716 (7.4) | 31 874 (9.9) | 12 955 (12.1) | 6 442 (14.0) | 99 987 (8.8) | 130 361 (7.4) |
| **Any of the above digestive system and related disorders** | **99 005 (16.0)** | **155 153 (23.5)** | **92 765 (28.7)** | **34 102 (31.9)** | **15 175 (32.9)** | **297 195 (26.1)** | **396 200 (22.6)** |

| **Urinary system disorders,** n (%) |  |  |  |  |  |  |  |
| --- | --- | --- | --- | --- | --- | --- | --- |
| Urinary incontinence | 33 118 (5.3) | 48 751 (7.4) | 32 196 (10.0) | 13 177 (12.3) | 6 584 (14.3) | 100 708 (8.9) | 133 826 (7.6) |
| Chronic kidney disease (CKD) |  |  |  |  |  |  |  |
| CKD 1 | 395 952 (63.9) | 359 133 (54.3) | 176 181 (54.5) | 61 953 (57.9) | 28 462 (61.7) | 625 729 (55.0) | 1 021 681 (58.2) |
| CKD 2 | 156 457 (25.3) | 220 878 (33.4) | 105 711 (32.7) | 31 309 (29.3) | 11 616 (25.2) | 369 514 (32.5) | 525 971 (29.9) |
| CKD 3A | 16 382 (2.6) | 25 226 (3.8) | 13 601 (4.2) | 4 489 (4.2) | 1 922 (4.2) | 45 238 (4.0) | 61 620 (3.5) |
| CKD 3B | 3 618 (0.6) | 5 141 (0.8) | 3 113 (1.0) | 1 168 (1.1) | 529 (1.1) | 9 951 (0.9) | 13 569 (0.8) |
| CKD 4 | 404 (0.1) | 532 (0.1) | 343 (0.1) | 136 (0.1) | 63 (0.1) | 1 074 (0.1) | 1 478 (0.1) |
| CKD 5ND | 15 (0.0) | 25 (0.0) | 9 (0.0) | 3 (0.0) | 2 (0.0) | 39 (0.0) | 54 (0.0) |
| Renal replacement therapy | 20 217 (3.3) | 32 643 (4.9) | 19 089 (5.9) | 6 764 (6.3) | 3 142 (6.8) | 61 638 (5.4) | 81 855 (4.7) |
| Missing data | 26 459 (4.3) | 17 553 (2.7) | 5 047 (1.6) | 1 095 (1.0) | 409 (0.9) | 24 104 (2.1) | 50 563 (2.9) |
| **Any of the above urinary system disorders** | **66 867 (10.8)** | **101 360 (15.3)** | **60 902 (18.8)** | **22 666 (21.2)** | **10 765 (23.3)** | **195 693 (17.2)** | **262 560 (14.9)** |
| **Respiratory disorders,** n (%) |  |  |  |  |  |  |  |
| Asthma | 52 387 (8.5) | 61 413 (9.3) | 37 017 (11.5) | 15 240 (14.3) | 8 097 (17.5) | 121 767 (10.7) | 174 154 (9.9) |
| Chronic obstructive pulmonary disease (COPD) | 32 382 (5.2) | 39 934 (6.0) | 24 832 (7.7) | 10 172 (9.5) | 5 461 (11.8) | 80 399 (7.1) | 112 781 (6.4) |
| Obstructive sleep apnoea (OSA) | 1 057 (0.2) | 3 522 (0.5) | 4 079 (1.3) | 2 708 (2.5) | 2 138 (4.6) | 12 447 (1.1) | 13 504 (0.8) |
| **Any of the above respiratory disorders** | **72 408 (11.7)** | **86 037 (13.0)** | **52 502 (16.2)** | **21 739 (20.3)** | **11 562 (25.1)** | **171 840 (15.1)** | **244 248 (13.9)** |
| **Neuro-psychological disorders,** n (%) |  |  |  |  |  |  |  |
| Depression - Medication-treated |  |  |  |  |  |  |  |
| One year before index date | 67 473 (10.9) | 82 266 (12.4) | 46 024 (14.2) | 16 764 (15.7) | 7 431 (16.1) | 152 485 (13.4) | 219 958 (12.5) |
| 5 years before index date | 114 375 (18.5) | 135 779 (20.5) | 75 051 (23.2) | 27 147 (25.4) | 11 994 (26.0) | 249 971 (22.0) | 364 346 (20.7) |
| **Ever** | **124 997 (20.2)** | **144 402 (21.8)** | **77 081 (23.9)** | **27 063 (25.3)** | **12 055 (26.1)** | **260 601 (22.9)** | **385 598 (21.9)** |
| **Musculo-skeletal disorders** |  |  |  |  |  |  |  |
| Osteoporosis | 74 170 (12.0) | 75 289 (11.4) | 35 633 (11.0) | 11 437 (10.7) | 4 374 (9.5) | 126 733 (11.1) | 200 903 (11.4) |
| Osteoarthritis | 63 288 (10.2) | 113 593 (17.2) | 78 101 (24.2) | 32 299 (30.2) | 16 031 (34.7) | 240 024 (21.1) | 303 312 (17.3) |
| **Any of the above Musculo-skeletal disorders** | **114 181 (18.4)** | **156 243 (23.6)** | **94 017 (29.1)** | **36 426 (34.1)** | **17 367 (37.6)** | **304 053 (26.7)** | **418 234 (23.8)** |
| **Malignancy** |  |  |  |  |  |  |  |
| Active malignancy – Any type of cancer | 26 677 (4.3) | 33 277 (5.0) | 16 832 (5.2) | 5 538 (5.2) | 2 360 (5.1) | 58 007 (5.1) | 84 684 (4.8) |
| Breast cancer (ever) | 11 488 (1.9) | 13 639 (2.1) | 7 748 (2.4) | 2 959 (2.8) | 1 139 (2.5) | 25 485 (2.2) | 36 973 (2.1) |
| Colorectal cancer (ever) | 6 742 (1.1) | 9 218 (1.4) | 4 709 (1.5) | 1 441 (1.3) | 540 (1.2) | 15 908 (1.4) | 22 650 (1.3) |
| Pancreatic cancer (ever) | 498 (0.1) | 456 (0.1) | 170 (0.1) | 61 (0.1) | 10 (0.0) | 697 (0.1) | 1 195 (0.1) |
| Endometrial cancer (ever) | 804 (0.1) | 1 414 (0.2) | 1 116 (0.3) | 640 (0.6) | 458 (1.0) | 3 628 (0.3) | 4 432 (0.3) |
| Ovarian cancer (ever) | 1 046 (0.2) | 1 130 (0.2) | 713 (0.2) | 282 (0.3) | 131 (0.3) | 2 256 (0.2) | 3 302 (0.2) |
| Esophageal cancer (ever) | 210 (0.0) | 144 (0.0) | 59 (0.0) | 18 (0.0) | 10 (0.0) | 231 (0.0) | 441 (0.0) |
| Kidney cancer (ever) | 1 465 (0.2) | 2 450 (0.4) | 1 403 (0.4) | 455 (0.4) | 178 (0.4) | 4 486 (0.4) | 5 951 (0.3) |
| **Any of the above malignancy types** | **37 998 (6.1)** | **48 093 (7.3)** | **24 978 (7.7)** | **8 445 (7.9)** | **3 553 (7.7)** | **85 069 (7.5)** | **123 067 (7.0)** |
| **Number of body system-related morbidity (BSRM)** |  |  |  |  |  |  |  |
| **0** | **208 214 (33.6)** | **127 667 (19.3)** | **39 375 (12.2)** | **9 715 (9.1)** | **3 609 (7.8)** | **180 366 (15.9)** | **388 580 (22.1)** |
| **1** | **151 290 (24.4)** | **140 614 (21.3)** | **57 316 (17.7)** | **16 490 (15.4)** | **6 613 (14.3)** | **221 033 (19.4)** | **372 323 (21.2)** |
| **2** | **101 791 (16.4)** | **137 532 (20.8)** | **70 114 (21.7)** | **22 391 (20.9)** | **8 996 (19.5)** | **239 033 (21.0)** | **340 824 (19.4)** |
| **3** | **70 404 (11.4)** | **110 675 (16.7)** | **62 895 (19.5)** | **21 653 (20.3)** | **9 394 (20.4)** | **204 617 (18.0)** | **275 021 (15.7)** |
| **4** | **47 028 (7.6)** | **76 693 (11.6)** | **47 185 (14.6)** | **17 537 (16.4)** | **8 001 (17.3)** | **149 416 (13.1)** | **196 444 (11.2)** |
| **5** | **26 697 (4.3)** | **43 953 (6.6)** | **29 303 (9.1)** | **11 692 (10.9)** | **5 591 (12.1)** | **90 539 (8.0)** | **117 236 (6.7)** |
| **6** | **11 015 (1.8)** | **18 742 (2.8)** | **13 005 (4.0)** | **5 679 (5.3)** | **3 023 (6.6)** | **40 449 (3.6)** | **51 464 (2.9)** |
| **7** | **2 739 (0.4)** | **4 766 (0.7)** | **3 504 (1.1)** | **1 575 (1.5)** | **814 (1.8)** | **10 659 (0.9)** | **13 398 (0.8)** |
| **8** | **326 (0.1)** | **489 (0.1)** | **397 (0.1)** | **185 (0.2)** | **104 (0.2)** | **1 175 (0.1)** | **1 501 (0.1)** |
| **Total, mean (SD)** | **1.57 (1.62)** | **2.15 (1.69)** | **2.56 (1.70)** | **2.82 (1.72)** | **2.98 (1.75)** | **2.37 (1.72)** | **2.09 (1.73)** |
| **Charlson score*** | **0.97 (1.70)** | **1.35 (1.91)** | **1.65 (2.06)** | **1.85 (2.16)** | **1.97 (2.21)** | **1.50 (2.00)** | **1.32 (1.92)** |
| **Annual care burden** |  |  |  |  |  |  |  |
| **% of total burden** | 30% | 37% | 21% | 8% | 4% | 71% | 100% |
| **Relative estimated annual care burden per capita** | **1.00** | **1.18** | **1.38** | **1.57** | **1.88** | **1.30** | **N/A** |

* BSRM score and Charlson score were found to be moderately positively correlated, r(1756789) = .65, *p* < .001.
